# Hepatic sinusoidal hemophagocytosis with and without hemophagocytic lymphohistiocytosis

**DOI:** 10.1371/journal.pone.0226899

**Published:** 2019-12-30

**Authors:** Jacqueline De Gottardi, Matteo Montani, Anne Angelillo-Scherrer, Alicia Rovo, Annalisa Berzigotti

**Affiliations:** 1 Hepatology, University Clinic of Visceral Surgery and Medicine, Inselspital, DBMR, University of Bern, Berne, Switzerland; 2 Institute of Pathology, University of Bern, Berne, Switzerland; 3 Department of Hematology, Inselspital, University of Bern, Berne, Switzerland; University of Southern California, UNITED STATES

## Abstract

**Background/Purpose:**

Hemophagocytic lymphohistiocytosis (HLH) is a rare, life threatening hyperinflammatory syndrome. Sinusoidal hemophagocytosis is occasionally observed on liver biopsy in patients who do not have clinical suspicion of HLH. We aimed at comparing the clinical characteristics and outcomes of patients with signs of hemophagocytosis on liver biopsy meeting and not meeting the HLH diagnostic criteria.

**Methods:**

We reviewed the clinical, laboratory features and outcomes of all adult patients consecutively admitted in our center between 08/2011 and 08/2017 presenting with liver histology showing sinusoidal hemophagocytosis and of critically ill patients presenting with severe liver disease in whom hemophagocytosis was histologically confirmed. The characteristics of patients fulfilling and not fulfilling the diagnostic criteria of HLH were compared.

**Results:**

We identified 12 cases (58% male, median age 61, 75% with a chronic underlying disease) with liver histology showing sinusoidal hemophagocytosis. All had at least some of the clinical features typically associated with HLH. Six were critical ill patients. In 4 cases with insufficient laboratory and clinical criteria, liver biopsy allowed to confirm the HLH diagnosis. Six patients died, of which four met the diagnostic criteria for HLH. Two patients with chronic liver disease died despite not fulfilling the diagnostic criteria of HLH.

**Conclusion:**

Hemophagocytosis on liver biopsy may contribute to confirming a diagnosis of HLH in suspected cases with indeterminate clinical and laboratory findings. Sinusoidal hemophagocytosis in patients with cirrhosis was associated with bad outcome.

## Introduction

Hemophagocytic lymphohistiocytosis (HLH), also known as hemophagocytic syndrome, is a rare, life threatening hyperinflammatory syndrome characterized by excessive activation of histiocytes and T lymphocytes leading to highly active but ineffective immune response [1]. HLH is classified into primary and secondary forms. Primary HLH is associated with different genetic defects in cytotoxic pathway that typically occur before age 18 but has been described in adults up to age 60 [2]. In contrast, secondary or acquired HLH is seen in adults who have no underlying family history, showing however other underlying conditions which can induce strong immunological stimulation, such as haematological or solid malignancies, infections, rheumatic diseases and autoimmune diseases [3]. Infections associated with HLH are most frequently caused by viruses (e.g. Epstein-Barr, Cytomegalovirus, Herpes simplex), but bacterial, fungal, and parasites have also been described [4]. HLH symptoms may initially mirror those observed in an infection (fever, malaise and fatigue). However, in patients with HLH the subsequent hyperinflammatory response is fatal in a high percentage of cases if left untreated (50–100% depending on the underlying condition) [1]. Diagnosing HLH is the critical step toward successful therapy, which is currently based on corticosteroids associated with other immunosuppressant drugs [5].

Despite increasing insights into its genetic and immunologic basis [5]], HLH is still diagnosed based on a combination of clinical and laboratory findings (Table 1).

**Table 1:** Diagnostic criteria for HLH used in the HLH-2004 trial*. The diagnosis of HLH is established when 5 of the 8 criteria listed below are fulfilled.

1. Fever	≥ 38.5°C
2. Splenomegaly	
3. Cytopenias (affecting at least 2 of 3 lineages in the peripheral blood)	Hemoglobin < 9 g/dL; Platelets < 100 x 10^3^/mL; Neutrophils < 1 x 10^3^/mL
4. Hypertriglyceridemia and/or hypofibrinogenemia	fasting, > 265 mg/dL <150 mg/dL
5. Hemophagocytosis	in bone marrow, spleen, lymph nodes, or liver
6. Low or absent NK-cell activity	
7. Ferritin	> 500 μg/L ‡
8. Elevated sCD25 (alpha-chain of sIL-2 receptor) §	

*modified from Henter et al. [6]]

Notes

‡Although the HLH-2004 protocol uses ferritin > 500 μg/L, higher values should prompt a higher clinical suspicion, namely ferritin > 3000 μg/L might be seen as suspicious of HLH and ferritin > 10 000 μg/L as highly suspicious for HLH.

§Elevations above age-adjusted, laboratory-specific normal levels (defined as > 2 SD from the mean) appear more meaningful than the original designation of >2400 U/mL because of variations between laboratories.

Many of these findings are nonspecific and are commonly observed systemic inflammatory response syndrome. In a landmark prospective international treatment study of HLH in children, the Histiocyte Society suggested five diagnostic criteria: fever, splenomegaly, bicytopenia (neutro- and thrombocytopenia), hypertriglyceridemia and/or hypofibrinogenemia, and hemophagocytosis in target organs (bone marrow, liver, spleen and lymph nodes) [7]. Subsequently, three additional criteria have been added [6]: hyperferritinemia, low/absent NK-cell-activity, and high-soluble interleukin-2-receptor (CD25) levels. Among the eight criteria, five must be fulfilled to diagnose HLH according to the HLH-2004 guidelines [6].

These criteria have been developed to diagnose HLH in children, but are usually adopted in adults as well since no specific study is available [8, 9]. In particular it has been suggested that ferritin levels above 10,000 μg /L are accurate for HLH diagnosis, and could be used to prompt further tests in adults [10]; this has been recently questioned [11] and hyperferritinemia alone should never be used to diagnose HLH. Special tests such as NK cell function (no longer done routinely) and soluble CD25 (alpha chain of the IL-2 receptor, marker of T-cell activation) as well as sCD163 (marker of macrophage activation) and CD107a (marker of degranulation) which more consistently correlated with the disease activity [9] are not available in all laboratories and cannot be usually immediately obtained. Therefore, treatment delay is frequent.

Despite the fact that elevated liver enzymes are found in 98% of cases of HLH [12], little is known regarding liver-related aspects of HLH in adults. Some cases presenting as acute hepatitis have been reported [13, 14], and occasionally, acute liver failure (ALF) may dominate the clinical presentation [15, 16].

Liver biopsy can reveal activated macrophages with hemophagocytosis (any hemopoietic cell included in the histiocyte, but most typically red cells and/or platelets) [17]. In primary, pediatric forms of HLH, liver histopathology shows a characteristic pattern with a portal and sinusoidal infiltrate of CD3+, CD8+, granzyme B+ lymphocytes admixed with CD68+, CD1a- (benign but activated) histiocytes that exhibit hemophagocytosis [18, 19]. Typically, endothelialitis of portal and central veins and lymphocyte-mediated bile duct injury is observed, and the degree of portal and sinusoidal lympho-histiocytic infiltrate and endothelialitis varies from mild to marked and correlates with clinical severity [17]. Interestingly, in one autoptic study conducted in children with primary HLH, bone marrow biopsy was often negative, while liver biopsy consistently showed “chronic hepatitis like” portal lymphocyte infiltrate, suggesting that findings at liver biopsy might be more sensitive for the diagnosis of HLH [19].

Given the disease’s nonspecific feature, its diagnosis in patients presenting with liver involvement is difficult and requires exclusion of more common conditions and histological confirmation.

On the other hand, in patients presenting with acute liver disease, but lacking the classical clinical diagnostic criteria of HLH syndrome, histological findings of sinusoidal hemophagocytosis are occasionally seen on liver biopsy and are considered non-specific for HLH [20], but the clinical relevance of these findings is largely unclear.

The purpose of this study is to describe the clinical features and outcomes of patients consecutively admitted in our center in the period 08/2011-08/2017 in whom liver histology showed sinusoidal hemophagocytosis (irrespective of whether HLH was eventually diagnosed). We aimed to compare patients’ clinical characteristics in those with and without secondary HLH according to the established HLH diagnostic criteria. Furthermore, we evaluated in how many of our adult cases the presence of sinusoidal hemophagocytosis on liver biopsy was a valuable additional diagnostic criterion.

## Materials and Methods

This was a single center, retrospective study conducted at Inselspital, University of Bern, Switzerland, in adult patients. This study has been approved by the Ethic Committee of the Canton of Bern (KEK-BE: 2017–00148) and informed consent was not required. All procedures followed were in accordance with the ethical standards of the responsible committee on human experimentation (institutional and national) and with the Helsinki Declaration of 1975, as revised in 2008. Cases were identified and collected in a 6 year period, from 08/2011 to 08/2017 by accessing the Pathology Institute of the University of Bern (electronic record system last accessed on August 10, 2017) identifying cases showing sinusoidal hemophagocytosis on liver histology; and by reviewing all adult patients with critically ill disease presenting with severe liver disease (MELD score [21] ≥15 and/or Child-Pugh class C) in whom HLH was histologically confirmed on any tissue (recorded at the hepatology unit of our center). Clinical, radiological and laboratory data were retrieved from the electronic record system of Inselspital (CGM Phoenix, Compu Group Medical Schweiz, AG). We retrospectively reviewed the patients’ medical records and retrieved information regarding their underlying diseases, possible triggers of hemophagocytosis, clinical features, laboratory data, treatment and outcome. We assessed the criteria recommended by the Histiocyte Society (Table 1) and calculated the Score of Saint Antoine (Hscore) [22] using the online calculator: http://saintantoine.aphp.fr/score/. Furthermore, we evaluated the presence of sinusoidal hemophagocytosis as possible additional diagnostic criterion to be used in adults. Soluble CD25 and NK were not quantified in the presented patients. On liver biopsy samples, the presence of sinusoidal hemophagocytosis was reviewed by a single experienced pathologist. Ascertained by haematoxylin and eosin (HE) staining; quantification of number of foci of hemophagocytosis per 20x field was performed. Immunohistochemistry CD68 staining was made to better identify activated macrophages. The number of CD68 positive macrophages per high power field was determined.

Cases of primary HLH were excluded from this analysis.

### Statistical analysis

Numerical data are described using median, interquartile range (IQR) and range, and percentages are given when required. Non-parametric tests were used to compare numerical parameters, and to obtain correlations between clinical and laboratory parameters and findings on histology. All tests are 2-sided and p<0.05 was considered significant. Statistical analysis was performed using PAWS statistics version 23 for Windows (SPSS Inc, Chicago, IL, USA).

## Results

We identified 12 cases with liver histology showing sinusoidal hemophagocytosis. Most of them were male (58%). The median age was 61 (IQR 26; range 22–81) years old. Seven patients (58%) were older than 70. Ten patients (83%) had an underlying chronic disease, which in 2 cases was diagnosed during the hospital admission.

Table 2 summarizes the clinical and laboratory features of the study population.

**Table 2:** Clinical, laboratory and histological characteristics of the included patients.

	HLH diagnostic criteria fulfilled before liver biopsy			HLH diagnostic criteria fulfilled on liver biopsy			Sinusoidal hemophagocytosis, but diagnostic criteria of HLH not fulfilled					
Case number	2	5	9	1	6	7	8	3	4	10	11	12
Gender, age (y/o)	Male, 71	Female, 73	Female, 70	Female, 22	Male, 47	Male, 74	Female, 75	Male, 52	Male, 60	Male, 24	Male, 81	Female, 77
**Underlying chronic disease**	Pancolitis interpreted as ulcerative colitis, on immunosuppressive therapy	Chronic lymphocitic leukemia and Evans Syndrome	Anaplastic lymphoma (diagnosed during the hospitalisation)	Systemic lupus erythemathodes and secondary antiphospholipid syndrome	None	Pure white cell aplasia	Evans Syndrome (Possible autoimmune hepatitis identified on biopsy)	Alcoholic cirrhosis and portal hypertension (large varices)	Evans syndrome/ granulomatous vasculitis; chronic liver disease with septal and porto-portal fibrosis (unclear origin)	None	T cell Lymphoma	Autoimmune hepatitis (diagnosed on liver biopsy during the hospitalisation)
**Possible infectious trigger for HLH**	CMV infection	None identified	S.aureus sepsis	Pancolitis of unclear origin	Bacterial pneumonia	Perianal abscess and bilateral pneumonia due to Pseudomonas aeruginosa	None identified	Suspected hemolytic-uremic syndrome	CMV infection	None identified	None identified	None identified
**Type of presentation**	High fever and pancytopenia + progressive increase of LFTs. Bone marrow biopsy negative.	High fever, pancytopenia and worsening of the general status in the last 3 weeks (following a bone marrow biopsy, negative)	Jaundice and MOF	High fever and pancytopenia, followed by spontaneous retroperitoneal hematoma on the second day of hospitalisation	High fever and increased LFTs since 1 week	High fever and pancytopenia + cholestasis interpreted as sepsis; three bone marrow biopsies in the weeks before hospitalisation were negative	Pancytopenia, high fever and elevated LFTs since 1 month; hepatosplenomegaly and lymphadenopathy; bone marrow biopsy negative	Diarrhea with lower GI bleeding, pancytopenia and coma interpreted as hepatic encephalopathy	Chronic elevation of LFTs and ascites	FUO, elevated liver enzymes	Jaundice and anemia	Jaundice and increased LFTs associated with polyarthralgias
									hemolytic anemia non responsive to high doses of prednisone			
**Severely ill patient**	Yes	Yes	Yes	Yes	No	Yes	No	Yes	No	No	No	No
***Clinical criteria of HLH***												
**Fever >38.5°**	+	+	+	+	+	+	+	─	─	+	─	─
**Splenomegaly**	+	+	+	─	+	+	+	+	+	+	─	─
**Bicytopenia**	+	+	+	+	─	+	+	+	─	─	─	─
**Hypertriglyceridemia and/or hypofibrinogenemia**	+	+	+	+	+	TG not measured/	TG not measured/	─	+	TG not measured/	─	─
						Fibrinogen normal	Fibrinogen normal			Fibrinogen normal		
**Hyperferritinemia**	+	+	+	+	+	+	+	+	+	+	+	+
**EBV Status**	IgG not performed, PCR negative	IgG positive, PCR negative	IgG positive, IgM negative	Not available	IgG positive, IgM negative	IgG positive, IgM negative	IgG positive, IgM negative, EBV in situ hybridization negative	IgG positive, IgM negative PCR negative	IgG positive, IgM negative	Not available	IgG positive, IgM negative	Not available
**Hemophagocytosis on histology**	+	+	+	+	+	+	+	+	+	+	+	+
**N criteria fulfilled before liver biopsy**	5	5	5	4	4	4	4	3	3	3	1	1
**N criteria fulfilled after liver biopsy**	6	6	6	5	5	5	5	4	4	4	2	2
***Other relevant clinical findings***												
**Ascites (none/moderate/severe)**	none	none	none	none	none	none	none	severe	moderate	N/A	moderate	none
***Biochemistry at presentation***												
**Hemoglobin, g/L**	70	73	89	77	135	75	63	78	71	129	77	147
**Platelet count, x 10**^**9**^**/L**	11	24	84	1	342	91	2	15	116	normal	263	143
**White cells (neutrophils), x 10**^**9**^**/L**	1	0.77	0.089	3.29	8.84	0.01	N/A	0.69	9.89	normal	4.2	5.08
**Serum ferritin, mg/L (15–300)**	16000	110000	18000	12684	1600	1590	733	4031	7380	1394	24905	9441
**AST/ALT, U/L (38/40)**	283/183	340/306	350/364	49/43	48/77	31/31	176/112	181/63	42/23	N/A	183/185	1187/2243
**Bilirubin, mmol/L (<17)**	66	19	17	4	36	40	127	270	98	N/A	1038	200
**ALP, U/L**	321	250	532	111	123	167	157	112	78	N/A	509	172
**GGT, U/L**	370	198	640	166	151	248	242	151	53	N/A	1105	227
**Albumin, G/L (>35)**	20	28	29	20	28	11	19	23	24	N/A	34	24
**INR (1.00)**	1.26	2.2	2.6	2.94	1	1.2	1.2	1.58	1.4	N/A	1.13	1.35
**Creatinine, mmol/L**	199	74	70	485	86	127	42	228	76	N/A	122	58
** Hscore**	236	221	192	228	119	127	132	190	169	90	167	102
***Biochemistry—maximal value***												
**AST/ALT, U/L (38/40)**	350/204	3914/1196	357/364	159/30	114/188	33/31	176/112	1966/1016	167/68	N/A	183/185	1319/2310
**Bilirubin, mmol/L (<17)**	293	56	186	58	36	343	127	270	1070	N/A	1038	260
**ALP, U/L**	321	425	532	197	323	263	157	384	78	N/A	509	172
**GGT, U/L**	370	599	640	483	322	293	242	151	53	N/A	1105	240
***Time from hospital admission to biopsy*, *days***	12	5	2	21	9	25	16	6	21	10	1	2
***N foci of hemophagocytosis in liver biopsy (per 20x magnification)***	15	20	20	22	8	10	15	N/A	11	22	N/A	50
**Sinusoidal dilatation**	absent	absent	absent	present	present	absent	absent	N/A	present	present	absent	absent
**Liver fibrosis**	absent	absent	absent	absent	absent	absent	low grade, non-septal	N/A	present, septal	absent	absent	absent
**Lobular necrosis**	present (centrolobular)	present (centrolobular)	absent	present (centrolobular)	absent	absent	present (intralobular)	N/A	absent	absent	absent	absent
**Portal infiltration**	plasma cell infiltrate	absent	absent	absent	absent	absent	Plasma cell infiltrate	N/A	absent	mixed cell infiltrate	absent	plasma cell infiltrate
***CD68***	62	45	N/A	91	73	74	48	N/A	59	78	N/A	75
***Specific therapy for HLH***	Yes	Yes	Yes	Yes	No	No	No	Yes	No	Unknown	No	No
***Time from hospital admission to HLH therapy initiation*, *days***	13	6	2	No HLH Spec. Rx	No HLH Spec. Rx	No HLH Spec. Rx	No HLH Spec. Rx	7	No HLH Spec. Rx	No HLH Spec. Rx	No HLH Spec. Rx	No HLH Spec. Rx
***Therapy received***	Etoposide and corticosteroids + Antibiotics (ciprofloxacin) + gancyclovir	Etoposide and corticosteroids + empirical gancyclovir and antibiotics	Etoposide and steroids + antibiotics	Corticosteroids and IL-1 antagonists (for the underlying lupus) + antibiotics	Antibiotics only	Antibiotics (imipenem/cilastatin) + cyclosporin for PRCA	Corticosteroids for Evans Syndrome	Plasmapheresis and corticosteroids + antibiotics	Corticosteroids and cyclosporin for underlying disease + gancyclovir	Unknown	None	Corticosteroids for AIH
											case interpreted as concomitant DILI	
***Outcome***	Progressive improvement; discharged after 30 days ICU; chronic slight AST/ALT elevation. Liver biopsy 1 year after discharge: signs of hemophagocytosis	Death due to MOF and DIC 12 days after presentation	Death 10 days after hospitalisation due to MOF	Progressive worsening with MOF; death 26 days after presentation and hospitalisation	Rapid remission; discharged after 7 days; no recurrence	Progressive worsening with development of jaundice and anasarca; therapy offered on histological confirmation of HLH, but patient chose not to be treated. Death 30 days after presentation (5 days after diagnosis)	Remission on steroids; no recurrence	Death 16 days after presentation (cardiac sudden death during ongoing MOF)	Spontaneous bacterial peritonitis and jaundice associated with hepatic encephalopathy grade 2. Death 36 days after presentation	Unknown	Progressive improvement, but still cholestatic after 3 months	Remission

In all cases the records showed a detailed workup of all relevant differential diagnoses, including sepsis (microbiology workup) and evaluation of the anemia (Coombs test, LDH, haptoglobin, unconjugated bilirubin, schistocytes).

Signs of sinusoidal hemophagocytosis detected on liver biopsy were rare in our center, being this finding seen in approximately 0.3% of all liver biopsies performed in the study period (n = 3855). However, seven of the cases were diagnosed in the last two years suggesting an increased awareness of HLH and the association of this pathologic finding to HLH.

Reasons for liver biopsy in our patients were increased acute or chronic liver function tests (n = 8), or fever of unknown origin associated with alteration of liver function tests.

### Clinical and laboratory features of patients meeting the HLH diagnostic criteria

Six patients (three males; median age 71 years; IQR 32; range 22–74) met the diagnostic criteria for HLH (Table 2); in three of them (50%), the HLH diagnosis was made after performing transjugular liver biopsy, upon the finding of sinusoidal hemophagocytosis. Interestingly, in two cases (case 6 and 7) the Hscore was low- namely 119 and 127- reflecting a <10% risk of HLH.

All patients but one were severely ill on admission, and all but one (patient 6, who was also not severely ill) had underlying chronic diseases: two had autoimmune diseases on chronic immunosuppressive therapy (ulcerative colitis in one; systemic lupus erythemathodes and secondary antiphospholipid syndrome in one) and three had hematological diseases (Chronic lymphocitic leukemia and Evans Syndrome in one; anaplastic lymphoma–that was diagnosed during the hospitalization- in one, and pure white cell aplasia in one). All patients but one (patient 9) presented with high fever, in four cases associates with pancytopenia. In three patients bone marrow biopsy was performed and did not show signs of hemophagocytosis. The most common diagnosis on admission was sepsis.

All cases showed increase in LFTs; usually, transaminases were moderately increased (2–10 times the upper limit of normality). All patients showed hypoalbuminemia. Jaundice was present in three patients on admission and developed in all cases but one during the course of the hospitalisation.

A possible infectious trigger for HLH was identified in 5 patients.

The median time interval between the hospital admission and liver biopsy was 11 days (IQR 18; range 2 to 25). The time from hospital admission to the histological diagnosis was significantly shorter in patients presenting with higher ferritin and higher alkaline phosphatase (ALP) (5 days vs. 21 days in median; p = 0.03).

All patients received antibiotics, two of them received additionally antivirals treatment (i.e. ganciclovir) aimed at controlling a suspected (in one case) or diagnosed (in another case) underlying CMV infection. Five patients received immunosuppressants (steroids and etoposide in three, steroids and anti-IL1 in one, cyclosporin in one). The immunosuppressive therapy was ongoing since the hospitalization in three cases, while a specific therapy was started in three patients respectively 2, 6 and 13 days after the admission, as soon as the report of the liver biopsy became available (on the same day or on the following day). Four patients (67%) died during the hospitalization, while two (case 2 and 6) recovered. One of the survivors (case 2) showed a chronic slight elevation of cholestasis enzymes on follow-up. A second liver biopsy was obtained one year after the first admission and showed again signs of sinusoidal hemophagocytosis.

### Clinical and laboratory features of patients not meeting the HLH diagnostic criteria

Six patients (4 males; median age 68 years; IQR 33; range 24–81) did not meet the diagnostic criteria for HLH (Table 2). The laboratory parameters on admission (AST, ALT, ALP, GGT, albumin, INR, creatinine, hemoglobin, platelet count) were similar to those of patients fulfilling the diagnostic criteria of HLH, and despite similar signs and symptoms, patients were less severely ill (only one patient was initially admitted to the ICU due to critical illness). Median Hscore was 149 points (IQR 75; range 90–190), with in median 3 criteria fulfilled (IQR 2; range 1–4). Similarly to patients fulfilling the diagnostic criteria of HLH, all patients but one had underlying chronic diseases: autoimmune conditions in three, hematological malignancy in one, and alcoholic cirrhosis with portal hypertension (large varices on endoscopy) in one; only this latter patient was severely ill on admission. One patient had a suspected hemolytic-uremic syndrome and one had a CMV infection as potential infectious triggers of their severe inflammatory status. In one case (Patient 8) in addition to sinusoidal hemophagocytosis findings on liver biopsy (fifth positive criterion for HLH), there were also signs suggesting autoimmune hepatitis. Bone marrow biopsy did not show signs of HLH. Furthermore, this patient showed a fast response to corticosteroids. The diagnosis of HLH was then considered unlikely. Three patients (one previously diagnosed with cirrhosis-case 3, and one with signs of advanced chronic liver disease on liver biopsy- case 4) had clinically evident, moderate ascites on presentation. All patients showed hypoalbuminemia and all patients were jaundiced on admission. Increase in transaminases was usually mild to moderate and very elevated values of AST or ALT (>15 times the upper limit of normality) were observed only in the patient diagnosed with autoimmune hepatitis (case 12) [23]. Two patients (33%), both with underlying advanced liver disease, became critically ill during the hospitalization and died. Four patients, including the two who died, received steroids as part of their treatment.

### Histological findings of liver biopsy, correlation with clinical presentation and outcome

Two patients with a definite diagnosis of HLH and two not fulfilling the criteria for HLH showed sinusoidal dilatation. None of the patients with HLH had fibrosis, while three of the patients not fulfilling the criteria for HLH showed some extent of liver fibrosis (non-septal in one, septal in two). Three patients with HLH showed centrolobular necrosis and one patient without HLH showed intralobular necrosis. A plasma cell infiltrate was observed in one patient with HLH, and in two patients without HLH; one additional patient without HLH showed mixed inflammatory cells infiltrate.

As far as the sinusoidal hemophagocytosis findings, histology and immunohistochemistry showed similar findings, irrespective of whether a final diagnosis of HLH was made.

**Fig 1:**
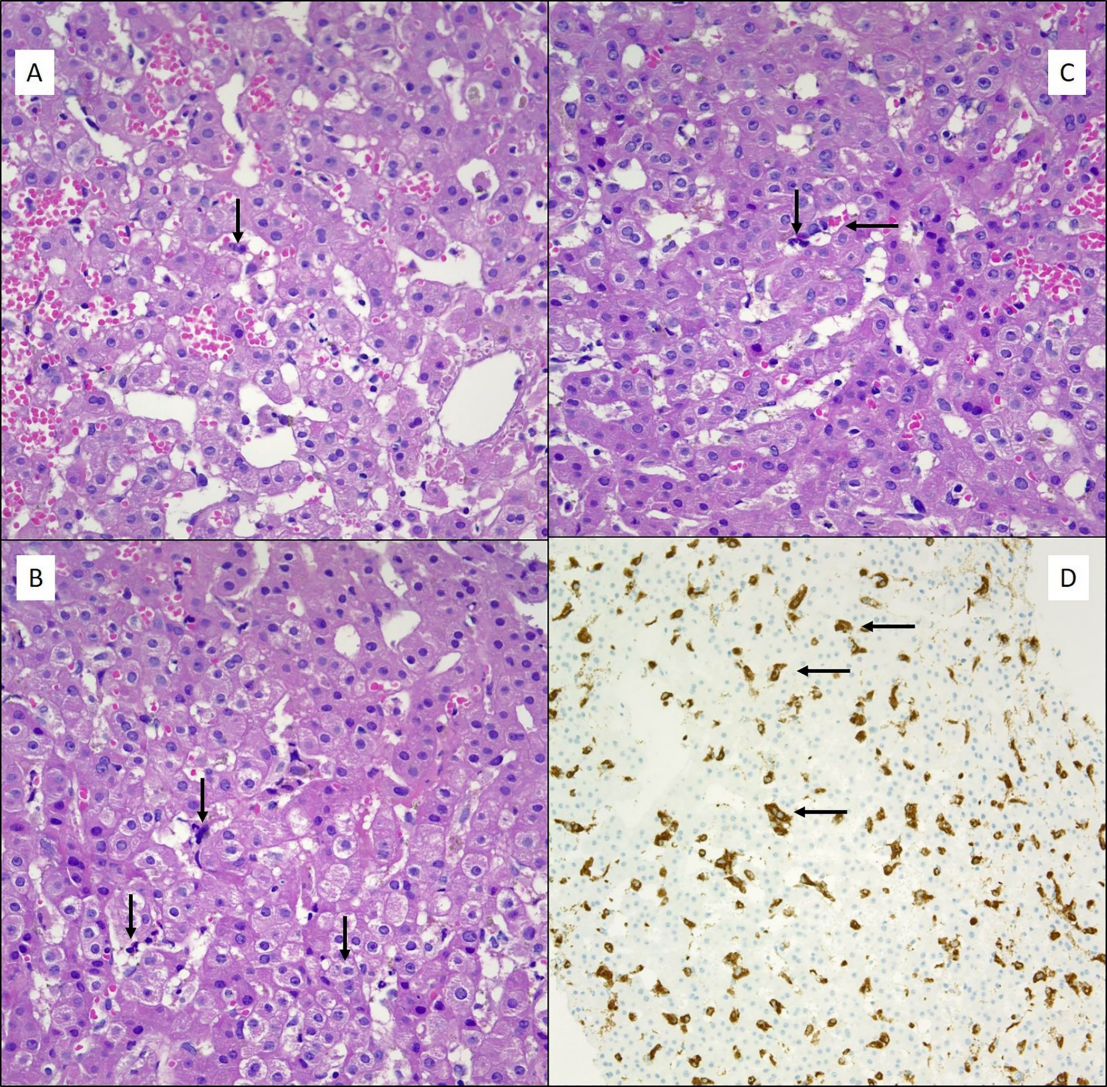
Liver biopsy showing signs of sinusoidal hemophagocytosis (arrows). Panel A and Panel B: HE (100x) patient with HLH syndrome. Panel C: HE (100x) patient fulfilling 3 diagnostic criteria of HLH syndrome. Panel D. IHC for CD68 (20x) showing multiple cells expressing CD68.

Fig 1 shows representative images HE staining revealed mononuclear cells infiltration in the portal tract and sinusoids.

Immunohistochemistry showed numerous CD68 positive macrophages, which, on HE staining, presented clear signs of hemophagocytosis. An exception was represented by a patient (case 12) presenting with features of autoimmune hepatitis associated with sinusoidal hemophagocytosis.

**Fig 2:**
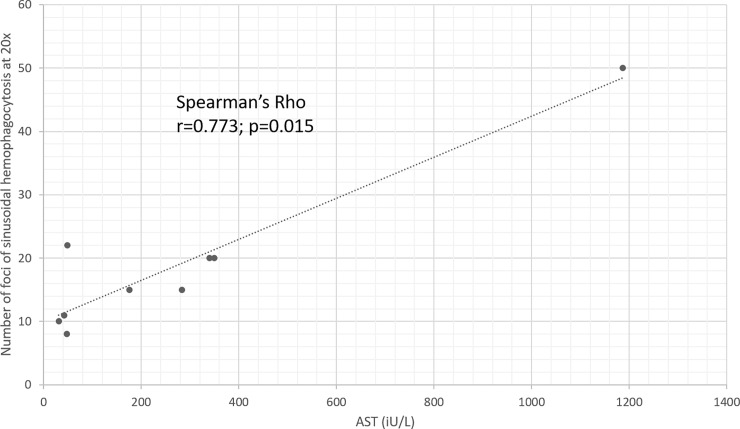
Correlation of AST and number of sinusoidal hemophagocytosis foci; observed at 20x on liver biopsy (Spearman’s Rho r = 0.773, p = 0.015).

Increased AST on presentation positively correlated with the number of foci of hemophagocytosis on liver biopsy (Spearman’s rho: 0.773, p = 0.015) (Fig 2).

### Mortality in patients with sinusoidal hemophagocytosis

As discussed above, in the present series of sinusoidal hemophagocytosis on liver biopsy, 50% (n = 6) died, mostly within one month since hospital admission (median 12 days; IQR 21; range 10 to 36). Irrespective of whether a final diagnosis of HLH was determined during admission, all patients who died, had severe underlying chronic diseases, and in all cases at admission a typical presentation including high fever, hepato-splenomegaly and bicytopenia was present (Table 2). With the limitation of a small number of events, none of the laboratory findings was statistically associated with fatal outcome, even if ferritin tended to be higher in patients who were deceased in comparison to survivors (median: 25’516 vs.10’536 μg/L).

## Discussion

Hemophagocytic lymphohistiocytosis (HLH), or haemophagocytic syndrome is a life-threatening disease with a reported mortality of over 50% [3], belonging to the so-called “cytokine storm syndromes”. In HLH, following a strong immunological stimulus, the uncontrolled proliferation of activated macrophages and lymphocytes leads to the secretion of a massive amount of inflammatory cytokines, which are thought to explain the organ damage [1]]. In patients with infection-triggered HLH, initial symptoms are non-specific, and the diagnosis of HLH can be challenging. [1, 4]]. Patients with HLH, however, cannot control the hyperinflammatory response which, if left untreated, is often fatal [1, 2, 24].

Despite its rarity, HLH is observed in up to 1% of patients with hematologic malignancy [24]] and has a dismal prognosis if left untreated. Clinical suspicion is key to drive the work-up recommended to diagnose HLH, which includes standard laboratory tests, specific tests and biopsy of organs that can be affected by the hemophagocytosis process.

In the largest series published so far in adult patients with HLH, alteration of LFTs is almost invariably observed [12, 25]], and AST/ALT elevation is considered among the criteria used to calculate the Hscore [22]]. Despite this, few cases of HLH presenting mainly as liver disease have been described in the literature [13, 14, 16, 25], and the significance of signs of hemophagocytosis observed in liver biopsy in patients not fulfilling the classical diagnostic criteria for HLH is currently unknown.

It has been previously reported that findings of hemophagocytosis in bone marrow aspirates is frequent even in subjects without HLH [26]. In our study focusing on liver biopsy, signs of hemophagocytosis were rarely found among all reviewed specimens. Our findings might suggest that the liver is less often involved, or later affected in the immunological processes leading to HLH. Given that liver biopsy is not frequently performed on suspicion of HLH, further studies with a larger number of patients would be valuable to confirm our findings. On the other hand, sinusoidal hemophagocytosis might be overlooked if not specifically sought; we observed an increase in the number of diagnosed cases at our center in the last two years, supporting an increased awareness for this sign and for HLH. It should be underlined that the finding of sinusoidal hemophagocytosis does not imply a diagnosis of HLH as this finding is considered non-specific and is present in other conditions characterized by immune activation such as sepsis [20]. In our series, half of the cases showing sinusoidal hemophagocytosis fulfilled the diagnostic criteria of HLH. Importantly, liver biopsy led to the definitive diagnosis of HLH in three patients in whom clinical and laboratory HLH diagnosis criteria were not otherwise fulfilled. It has to be taken into account that the diagnostic performance of Hscore in adults is debated [27]. However, a correct diagnosis ruling-out or ruling-in HLH is key, on one side to avoid immunosuppression when it is not needed, on the other side since newer therapies such as interferon gamma inhibitors may make a difference to outcomes in the future. The finding of sinusoidal hemophagocytosis can be considered an important diagnostic feature to be searched in adults upon clinical suspicion of HLH and indeterminate laboratory findings, and we suggest that transjugular liver biopsy can be considered as a further diagnostic tool to provide an additional indicator supporting HLH diagnosis and complementing clinical acumen and laboratory tests. In this regard, it should be underlined that transjugular liver biopsy is a safe procedure, even in patients with severe thrombocytopenia and coagulopathy [28]. We acknowledge that since liver biopsy is not routinely performed in patients already diagnosed with HLH at our center, we cannot provide data regarding the sensitivity of liver biopsy in the context of HLH, meaning that we do not know if patients with HLH might show negative results (no sinusoidal hemophagocytosis) on liver biopsy.

As for the patients showing sinusoidal hemophagocytosis but not fulfilling the diagnostic criteria for HLH, the clinical presentation was usually less severe as compared to patients fulfilling the criteria for HLH. On the other hand, both groups shared several features, including similar values of laboratory tests, and presence of underlying chronic diseases frequently associated with HLH [1, 6, 7]. This suggests that sinusoidal hemophagocytosis in the absence of HLH might indicate uncontrolled hepatic/systemic inflammation, leading to clinical features closely resembling HLH.

Regarding liver-related findings, most of the patients showed a mild to moderate increase of AST/ALT. A novel, interesting finding of the present study is that AST at presentation closely correlated with the severity of histological signs of hemophagocytosis, suggesting that monitoring AST on treatment might help in evaluating the inflammatory activityitself, and potentially in assessing treatment efficacy. Confirmation of this correlation in a larger number of patients would be of interest.

All our patients showed hypoalbuminemia. In our view, even if this is a non-specific finding, it should be included among those appearing in adult HLH, similarly to what is already done in children suspected of HLH[29].

Similarly to what occurs in patients with sepsis, jaundice (total bilirubin > 50μmol/L) was present at admission or developed over the course of the hospital admission in all patients with signs of hemophagocytosis but one, likely reflecting the effects of pro-inflammatory cytokines on several steps of bile flow (down regulation of bile acid transporters [30]; oxidative stress leading to the inhibition of cAMP-dependent transport function [31] eventually leading to intrahepatic cholestasis.

Interestingly, the number of CD68 macrophages on histology did not correlate with the number of positive foci. Consequently, the number of CD68 positive cells does not seem useful to demonstrate liver involvement in hemophagocytosis, which relies mostly on haematoxylin and eosin.

In agreement with previous reports regarding a very high mortality in patients with HLH [1, 12, 24], four patients with HLH included in our series died, all of them within approximately one month after being hospitalized. Similarly to the report by Li et al [12], all patients who died in our series presented typically with high fever, hepato-splenomegaly and bicytopenia, which should be considered as ominous prognostic signs. In addition, two patients with sinusoidal hemophagocytosis not fulfilling the diagnostic criteria for HLH died.

These patients had either a known cirrhosis or advanced liver disease on liver biopsy and presented with ascites. This suggest that in patients with cirrhosis—in whom immune-response is altered [32, 33]—hemophagocytosis likely indicates a particularly severe inflammatory activation, which might explain a very severe acute- on- chronic liver failure.

A novel observation in our series concerns the finding of signs of hemophagocytosis in a follow-up liver biopsy obtained one year after discharge in the patient surviving a first episode of HLH (triggered by viral and bacterial infection; case 2). Interestingly, bone marrow biopsy at the time of first diagnosis was negative. To our knowledge, chronic secondary hemophagocytosis in the liver has never been reported so far. Whether this might be linked to a genetic component with a liver-specific behavior is currently unknown.

In conclusion, signs of hemophagocytosis on liver biopsy can be found in patients fulfilling or not the diagnostic criteria for HLH. Mortality in HLH was very high in this series. In subjects in whom HLH is suspected transjugular liver biopsy might be considered since it can provide findings supporting a HLH diagnosis complementing clinical acumen and laboratory tests. Patients not fulfilling the diagnostic criteria for HLH showed similar risk factors and triggers as patients with HLH and two of them had a fatal outcome, suggesting that sinusoidal hemophagocytosis in the absence of HLH might still indicate uncontrolled hepatic and systemic inflammation, leading to clinical features closely resembling HLH. Signs of hemophagocytosis on histology in patients with a history of chronic liver disease seem to be an ominous prognostic sign even in the absence of HLH. Irrespective of whether a diagnosis of HLH is eventually made, all patients with suspected HLH and patients with findings of sinusoidal hemophagocytosis on liver biopsy should receive an extensive work-up aimed at identifying possible triggers amenable to treatment.

## Abbreviations

HLH: hemophagocytic lymphohistiocytosis
EBV: Epstein-Barr virus
CMV: cytomegalovirus
HIV: human immunodeficiency virus
ALF: acute liver failure
LT: liver transplantation
MOF: multi-organ failure
ACLF: acute on chronic liver failure
